# Progress in Medicine

**Published:** 1896-09-19

**Authors:** 


### Progress in Medicine.

### DISEASES OF THE LIVER.

**The Effects of Alcohol.**—Kerr[1] writes an interesting paper upon the effects of alcohol upon the liver. He points out the want of accord in the views of various writers upon the nature of the changes that follow its abuse. All agree that both enlargement and diminution in size may occur, but there is a difference of opinion as to whether these are distinct varieties, or as to whether a large liver may contract down in later stages to a small one. Some hold that the microscopic appearances differ in the two forms, while others do not consider that any hard and fast line can be drawn. The ill-defined state of medical knowledge upon these points is well known, but possibly it may be a surprise to some readers of Dr. Kerr's paper to find that it is doubtful whether cirrhosis can be said to be the most important deleterious result of the abuse of alcohol upon the liver. Alcohol affects not only the fibrous tissue of the gland, but the liver cells also, and these cells may suffer apart from any increase in the interstitial tissue. Kerr records two such cases, which he says may be considered examples of what Lancereaux has called steatosis of the liver. This condition he thinks must be of common occurrence, but has not attracted much attention in this country. In other cases examined by Kerr, although there was increase of embryonic tissue in the portal spaces of the liver, it was insignificant compared with the fatty changes in the liver cells. Another interesting point, and one that is more or less an illustration of the above facts, is the greater frequency of enlargement than of diminution in the size of the liver. Thus, of eighteen cases of disease of the liver following abuse of alcohol, eleven were above the normal in weight, and only four below, while three were about normal. One other fact is worthy of note. In only four of the above eighteen cases was the condition of the liver the cause of death, and in these four cases it was hæmatemesis. Foxwell[2] writes upon one of the above-mentioned points. He gives records of clinical cases and others from the post-mortem room to show that enlargement of the liver due to cirrhosis is a more common sequence of alcoholic intemperance than atrophy. Dr. Foxwell's cases which came to an autopsy are only eight in number, and these cannot be said to very strongly support his contention, since four were under the normal in weight, and only the same number above it. Dr. Foxwell, however, has probably only included those cases in which there was definite evidence of cirrhosis, and has omitted those in which alcohol had produced fatty degeneration of the liver cells in excess of increase in the interstitial tissue. Two cases of hypertrophic cirrhosis of Hanot occurring in brothers are described by Dreschfield.[3] In both cases there was considerable enlargement of the spleen, and some jaundice. Upon one case a post-mortem examination was made, and the spleen was found to weigh fifty ounces. Notes of another case are given by Potain,[4] and of a fourth by Weber.[5] In both of these there was jaundice, and in the case of Weber the spleen weighed 20 oz. Dreschfield considers that alcohol may have played a part in the etiology of the enlargement of the liver in one of his cases, and Weber thinks that the cirrhosis in his case was secondary to impaction of gall-stones in the common bile duct, while Potain attributes the disease to absorption of toxins from the intestine, combined with nervous worry. Krawkow[6] has contributed experiments which seem to support the view that cirrhosis of the liver may be set up by the absorption of toxins from the intestine. Large quantities of micro-organisms introduced into the alimentary canal of fowls gave rise to cirrhosis of the liver. It is mentioned that the fact that the absorption of putrid material from the intestine may give rise to cirrhosis suggests that alcohol does not act directly as a poison upon the liver substance, but by setting up intestinal catarrh facilitates the absorption of toxins. Gauthier[7] attributes the most important complication of hepatic cirrhosis, namely, gastro-intestinal hæmorrhage, to the influence in part of toxins. He considers that toxic substances unaltered by the liver are eliminated by the intestines. These lead to hæmorrhage, by causing dilatation of the gastro-intestinal vessels through their action on the ganglionic system.

**Cancer of the Gall-bladder.**—Kelynack[8] has investigated subject of the association of gall-stones and cancer of the gall-bladder, and comes to the conclusion that, although primary cancer of the gall-bladder may occur unassociated with the presence of gall-stones, in the large majority of cases the two exist together, and that it is reasonable to suppose in some cases at least that development of the malignant growth is favoured by the irritation of the calculi. A paper by Rolleston[9] upon the rarer disease of primary carcinoma of the bile ducts seems to show that in such cases gall-stones are less common. In eleven cases gall-stones were only present in four. Carcinoma in this situation is generally of the cylindrical-celled variety. It is interesting pathologically, and Rolleston seems to think its recognition clinically may be of some importance, since it would be more amenable to operative treatment than malignant disease in other situations causing jaundice. He is obliged to admit, however, that the diagnosis between carcinoma of the common bile duct, and of the head of the pancreas, can hardly be made. A case of primary sarcoma of the liver is recorded by Byron Bramwell[10] where a large blood cyst in its interior simulated a hepatic abscess and was opened and drained. The patient died, and the liver was found enormously enlarged by a tumour which had destroyed nearly the whole organ. It weighed 9 lbs. 6 ozs.

**Hœmatagenous Jaundice.**—Auld[11] does not think that the idea of hæmatagenous jaundice can be altogether put on one side. He confirms the observations of Hunter, which tend to show that toluylendiamin causes obstructive jaundice by producing viscidity of the bile and catarrh of the bile ducts; but he considers that the jaundice which follows poisoning by phenylhydrazin and metaphenylendiamin may have a different origin. These substances lead to great blood destruction, which apparently takes place chiefly in the spleen, where great formation of pigment occurs. A great deal of this pigment finds its way to the liver, where the products of blood destruction become eliminated. When the amount of poison given has been large the action of the liver cells becomes impaired, and some of the pigment finds its way into the general circulation. The pigments derived from broken-down blood cells, however, are not bile pigments, but Auld thinks that it is possible to conceive that the liver cells may be able to transform the free hæmoglobin into bile pigment, and yet not prevent its entrance into the general circulation. Plowright[12] refers to an epidemic of jaundice which occurred in King's Lynn in 1895. Thirty-four cases occurred in seven months. The majority occurred under the age of 30. In twenty-one cases there was more or less epigastric pain, and in twenty-four vomiting ushered in the jaundice. The majority were well at the end of ten days. No fatal case occurred.

**Carbohydrate Food.**—Paton's[13] experiments seem to show that carbohydrate food increases the fats in the liver. He thinks that these fats may be formed from glycogen. Proteid food does not lead to an accumulation of fats in the liver. Paton considers that, should it be proved that glycogen may be a source of fat, it does not follow that sugar may not be derived from this substance, as Pavy believes. Pavy[14] read a paper recently before the Physiological Society upon this subject, and gave details of experiments which he considers show that the formation of sugar from glycogen occurs only after death, and not during life.

[1] Med. Chronicle. Jan., 1896. [2] Birmingham Med. Review, April 1896. [3] Medical Chronicle, April, 1896. [4] Med. Week., May 8, 1896. [5] Brit. Med. Journal, April 25, 1896. [6] Archives de Med. Expériment, 1896, Nos. 1 and 2; Med. Chronicle, June, 1896; Brit. Med. Journal, March 28, 1896. [7] Journal de Med., March 10, 1896. [8] Practitioner, April, 1896. [9] Med. Chronicle, Jan., 1896. [10] Brit. Med. Journal, June 12, 1896. [11] Brit. Med. Journal, Jan. 18, 1896. [12] Brit. Med. Journ., May 30, 1896. [13] Edinburgh Med. Journal, May, 1896. [14] Lancet, July 8, 1896.

